# Acceptability of an mHealth App for Youth With Substance Use and Mental Health Needs: Iterative, Mixed Methods Design

**DOI:** 10.2196/30268

**Published:** 2021-12-24

**Authors:** Zachary Adams, Miyah Grant, Samantha Hupp, Taylor Scott, Amanda Feagans, Meredith Lois Phillips, Kristina Bixler, Phani Teja Nallam, Dorothy La Putt

**Affiliations:** 1 Department of Psychiatry Indiana University School of Medicine Indianapolis, IN United States; 2 Department of Psychology University of Indianapolis Indianapolis, IN United States; 3 Department of Psychiatry Indiana University-Purdue University Indianapolis Indianapolis, IN United States

**Keywords:** mobile health, user-centered design, adolescents, substance use disorders, mental health, mHealth, cognitive behavioral therapy, homework, technology acceptance model, trauma, mobile phone

## Abstract

**Background:**

Treating substance use disorders (SUDs) during adolescence can prevent adult addiction and improve youth outcomes. However, it can be challenging to keep adolescents with SUDs engaged in ongoing services, thus limiting potential benefits. Developmentally appropriate tools are needed to improve treatment engagement during and between sessions for youth with SUDs and mental health disorders. Mobile health apps may augment or replace psychotherapy components; however, few have been developed specifically for youth with SUDs following user-guided design principles, which may limit their appropriateness and utility. Formative research on acceptability to intended end users is needed before the efficacy of such tools can be examined.

**Objective:**

This study involves user-centered, iterative development and initial user testing of a web-based app for adolescents with SUDs and mental health concerns.

**Methods:**

Adolescents aged 14 to 17 years with past-year involvement in outpatient psychotherapy and behavioral health clinicians with adolescent SUD treatment caseloads were recruited. Across 2 assessment phases, 40 participants (alpha: 10 youths and 10 clinicians; beta: 10 youths and 10 clinicians) viewed an app demonstration and completed semistructured interviews and questionnaires about app content and functionality.

**Results:**

Participants expressed positive impressions of the app and its potential utility in augmenting outpatient therapy for youth with SUDs and mental health concerns. Noted strengths included valuable educational content, useful embedded resources, and a variety of activities. Adolescents and clinicians favored the app over conventional (paper-and-pencil) modalities, citing convenience and familiarity. The app was found to be user-friendly and likely to improve treatment engagement. Adolescents suggested the inclusion of privacy settings, and clinicians recommended more detailed instructions and simplified language.

**Conclusions:**

The novel app developed here appears to be a promising, acceptable, and highly scalable resource to support adolescents with SUDs and mental health concerns. Future studies should test the efficacy of such apps in enhancing adolescent behavioral health treatment engagement and outcomes.

## Introduction

### Background

Co-occurring substance use and mental health disorders are common in adolescence. Approximately 916,000 (3.7%) adolescents aged 12 to 17 years in the United States meet the criteria for ≥1 substance use disorder (SUD) [1]. Approximately 38% of 12th graders experimented with illicit drug use within the past year, whereas 47% reported using illicit drugs at least once during their lifetime [2]. Compared with adults, adolescents with SUDs are more likely to have a co-occurring mental health disorder [3], with 60% to 75% of adolescents exhibiting SUD meeting criteria for ≥1 disruptive behavior disorders, mood disorders, anxiety disorders, traumatic stress disorders, or other psychiatric disorders [1,4,5].

Despite the high rates of behavioral health disorders among youth, few receive appropriate health treatment services. In 2018, only 1 in 10 youths with SUDs received behavioral health treatment [6]. Individuals with co-occurring disorders typically experience greater symptom severity, require greater service use throughout treatment, and demonstrate poorer treatment outcomes compared with youth with single disorders [7,8]. Although treatment interventions for adolescents with SUDs show promising long-term outcomes, approximately 40% of adolescents discontinue prematurely before completing their treatments [9]. Collectively, these trends point to a need for better strategies for improving youth engagement in treatment services.

An additional barrier to care is that few intervention resources have been developed that specifically address improving care for youth with comorbid disorders [10]. Moreover, clinical service systems are often disjointed and address substance use and mental health separately [11]. Treatments meant to address co-occurring disorders often incorporate well-established, evidence-based principles often rooted in a cognitive behavioral therapy (CBT) framework. Such treatments emphasize the importance of identifying specific goals, understanding the antecedents and consequences of substance use, teaching new coping skills (eg, substance refusal, emotion regulation and relaxation, communication skills, and cognitive restructuring), progress monitoring, and practicing strategies in and between sessions [12-14]. Increasing patient involvement in homework exercises may be especially valuable, as more consistent homework completion in CBT is associated with greater skill mastery and more improvements in key clinical domains [14-16].

These components can be challenging to implement in conventional treatment contexts. For instance, youth often struggle to complete homework tasks as recommended in CBT [15,17]. Moreover, many clinicians lack confidence in treating co-occurring disorders, which can reduce fidelity to best practice models [18]. Thus, there is a need for scalable, sustainable resources that are not only engaging for youth but also helpful for providers in delivering evidence-based interventions to this population in line with best practice guidelines and principles.

Mobile health (mHealth) solutions that leverage remote functionality of smartphones and other devices are an increasingly feasible strategy for improving behavioral health treatments for adolescents [19], especially given the very high smartphone ownership and accessibility among youth, with 95% of teenagers reporting access to smartphone devices in 2019 [20]. There is also evidence that adolescents are open to using smartphone-delivered tools to support recovery and relapse prevention as part of SUD treatment [21]. Such tools, whether delivered via mobile apps or SMS text messaging, have shown promise in improving treatment adherence and engagement while also expanding access to care [22-24]. mHealth solutions can directly address the key treatment components of CBT. For instance, regarding homework, apps may send automated calendar reminders, present individualized guidance and educational content, and offer real-time feedback to users about their responses or behavior in ways that complement clinician-delivered content [19,25]. Although a growing number of mobile apps have emerged for mental and physical health, few are specifically designed to address the needs and preferences of adolescents with SUDs and co-occurring disorders [23,26].

When developing mHealth apps, the current best practice is to follow a user-centered, iterative design process to optimize usability and effectiveness for intended end users [27-29]. In this framework, both users and experts test the app and provide feedback on its relevance and functionality, which allows the tool to be tailored to the user’s needs while still adhering to theoretically or empirically supported principles [28]. This approach can provide valuable insight into which features are most important to users, such as personalization, autonomy, simplicity, and informativeness [30]. This process often involves a mixed methods approach to assessment, whereby users interact with the app or view a demonstration and are then given opportunities to share their reactions about the design, features, and functions of the app via standardized survey instruments and interviews.

As the goal of mHealth interventions is to allow users to change their behavior, both in terms of SUD and mental health–related behaviors but also with respect to using the app and adhering to treatment recommendations, the app development and evaluation process may also be guided by prominent behavior change theories. One such framework is an extension of the theory of reasoned action called the technology acceptance model (TAM), which posits that acceptance and attitude toward technology are directly influenced by perceived usefulness (PU) and perceived ease of use (PEOU) in achieving the intended goal (eg, delivering key treatment components such as homework activities or symptom monitoring) [31]. Such formative work to determine the acceptability and appropriateness of an app is critical before later tests of efficacy and effectiveness in improving clinical outcomes.

### Objective

The purpose of this study is to develop and perform initial usability pilot-testing of a mobile app designed to augment outpatient behavioral health treatment for adolescents with SUDs and co-occurring mental health concerns. Interactive content was rooted in evidence-based treatment principles, designed to appeal to youth, and evaluated following an iterative, user-centered design strategy in which both adolescents with SUDs and clinicians who treat youth with SUDs were involved to maximize relevance to treatment stakeholders.

## Methods

### Recruitment

Participants were recruited for two phases of this formative acceptability study: alpha testing (10 adolescents and 10 clinicians) and beta testing (10 youths and 10 clinicians). This sample size was initially selected to help ensure the collection of diverse reactions to the app from the intended end user groups at different points in the app development process. Participants viewed an app demonstration and completed semistructured interviews and questionnaires about app content and functionality.

Adolescent participants aged 14 to 17 years (alpha: mean 15.3, SD 1.0 years, 6/10, 60% girls and 3/10, 30% boys; beta: mean 15.9, SD 1.0 years, 8/10, 80% girls and 2/10, 20% boys) had current or past-year involvement in outpatient psychotherapy for ≥1 SUDs per self- and caregiver-report at the time of eligibility phone screening. Car, relax, alone, forget, friends, and trouble substance use screeners were also administered during phone screening to verify likely SUD status (all participants scored ≥1, consistent with published cutoff recommendations) [32-34]. Comprehensive diagnostic evaluations were outside the scope of this study and were not performed.

Clinician participants were eligible if they had an active adolescent SUD treatment caseload (range: alpha, 20%-100% of caseload; beta, 10%-100% of caseload) and had worked with ≥3 adolescents with co-occurring SUDs and mental health conditions in the past 5 years. Clinician participants (all female; age—alpha: mean 43.3, SD 7.5 years; beta*:* mean 37.8, SD 12.1 years) reported varied credentials (licensed clinical social worker: 6/20, 30%; licensed social worker: 4/20, 20%; licensed medical health counselor: 3/20, 15%; PhD: 2/20, 10%; PsyD: 2/20, 10%; other: 3/20, 15%) and years of experience (mean 11.6, SD 7.5 years). Both adolescents and clinicians were recruited from community and mental health and SUD treatment facilities, academic health system clinical research registries, community advertisements, and word of mouth. The procedures were approved by the university institutional review board. All participants completed consent and assent procedures during the enrollment process.

### mHealth App: Bright Path

**Figure 1:**
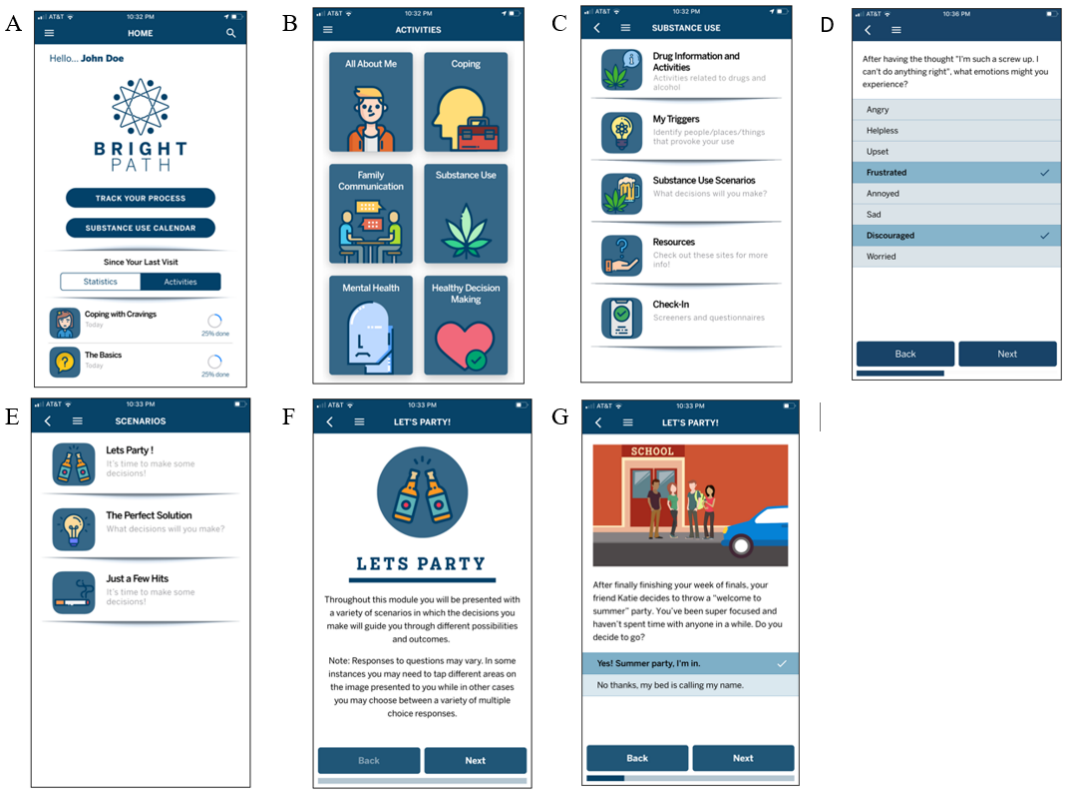
Screenshots from the Bright Path app. (A) Home screen with progress monitoring dashboard; (B) main menu of content topics; (C) first page of substance use–related activities menu; (D) sample of multiselect items included in an interactive coping exercise; (E) menu of scenario activities where users are instructed to read vignettes and make choices that advance the story; each choice either increases or decreases risk for substance use or negative health outcomes; (F) sample instruction screen from a scenario practice activity; (G) sample screen from a scenario practice activity with illustration and text accompanied by response choices. Feedback is presented to users based on their selections, and the story advances.

The novel, web-based mHealth app developed here was designed to present educational content and interactive games and activities that address common factors associated with substance use and mental health disorders in youth. The content is arranged into several sections (coping skills, substance use, mental health, family communication, and healthy decision-making), which are accessible from a menu screen. Within each section, users are presented with a menu of activities to choose from. For instance, within the *substance use* content area, users can access activities where they can perform tasks such as identifying their personal cues and triggers for substance use, completing substance use assessments (screening tools and self-report of recent substance use), practicing safe decisions in high-risk hypothetical scenarios, or learning about the effects of different types of substances. Within the *coping* section, activities address cognitive behavioral skills such as challenging automatic thoughts, selecting and scheduling prosocial activities, and learning about connections between thoughts, feelings, and behaviors. Throughout the app, each activity begins with basic instructions to help users know how to interact with the content. Several activities also include feedback slides that provide additional information about correct and incorrect answers or provide encouragement for continued progress. The app also includes an *all about me* section where users can enter basic information about themselves and customize certain features such as their name and picture. Activities are presented in various formats. Some are presented as surveys, some as web-based *card decks* (ie, question on one side and answer on the other side), and some as web-based *choose your own adventure* style scenarios. Figure 1 presents screenshots from the app depicting the general look and feel as well as the organization of the app.

### Measures

#### Qualitative Interview

In both the alpha and beta phases of the study, the mobile app *e-tools* were demonstrated in person on smartphones or through videoconferencing software. A semistructured interview was conducted using the think-aloud technique, a technique that has been routinely and successfully implemented in the usability evaluations of other web-based interventions [35,36]. Participants’ responses as they were guided through the app were audio recorded for transcription and analysis following the visit. Participants reviewed 1 of 2 subsets of approximately 8 interactive components within the app. Each subset component was selected on an alternating basis whereby half the participants viewed the first subset, and the other half viewed the second subset. An average of 5 to 10 minutes was dedicated to reviewing each component.

#### Quantitative Rating Instruments

Assessment and refinement of the mobile app were guided by the TAM, an extension of the theory of reasoned action. The two key factors emphasized by the TAM are (1) PU, which is the degree to which tools will accomplish the goal of enhancing treatment, and (2) PEOU, which encompasses the ease of navigation, technical problems, and reactions to the overall *look and feel* of the interface. PU and PEOU are theorized to predict actual system use through the measurement of attitudes toward use and behavioral intentions to use. Additional items assessed perceived ease of learning and overall satisfaction. A 46-item questionnaire administered to both youth and clinicians also assessed respondents’ perceived self-efficacy when using mobile devices as well as concerns related to the use of technology in treatment (eg, privacy and social acceptability). Subscales measuring PU (13 items; sample item: Using this app in treatment would help my patients accomplish tasks and goals more quickly), PEOU (14 items; sample item: I would find it easy to get this app to do what I want it to do), perceived ease of learning (4 items; sample item: It would be easy for me to become skillful using this app), and satisfaction (14 items; sample item: Using the app is a good idea) were calculated. These measures demonstrated internal consistency (PU, providers *α*=.89, youth *α*=.83; PEOU, providers *α*=.88, youth *α*=.92; perceived ease of learning, providers *α*=.87, youth *α*=.81; and satisfaction, 14 items, providers *α*=.86, youth *α*=.87).

#### Name and Logo Design

During the alpha phase of the study, youth and providers were presented with an array of 16 potential logos and app names generated through a series of brainstorming sessions within the clinical research team and in partnership with experts in graphic design. Participants were directed to select and rank their *top 5* preferred logo options. The most highly rated name (Bright Path) and logo across the youth and clinician participants were then selected and used throughout the beta phase of the study. The logo is depicted in Figure 1.

### Statistical Analysis

Qualitative data obtained during the interviews were transcribed and summarized. A total of 2 independent reviewers analyzed and interpreted the transcriptions, identifying and coding for common themes across participants and extracting illustrative quotes for each theme. Participant feedback that was collected during alpha testing guided refinements for beta testing to improve PU and PEOU. Mann–Whitney *U* tests were performed to test the hypothesis that PU and PEOU scores at beta testing were higher than PU and PEOU at alpha testing (ie, later version hypothesized to be more useful and easier to use than the earlier version). Similar analyses were performed comparing the alpha and beta testing groups on perceived ease of learning and satisfaction.

## Results

### Overview

Qualitative data from individual interviews with youth and providers were reviewed, and content was organized into four main themes: app content, user experience, app use in combination with outpatient therapy, and suggested app modifications. Specific examples quoted from participant interviews are presented in Table 1 to illustrate each of these categories as well as subordinate themes, which are summarized here.

**Table 1:** Common themes and illustrative quotations from formative interviews with youths and providers during alpha and beta testing demonstration sessions.

Domain and common themes		Participation interview quotes
**App content**		
	Valuable information for teens	“Having a structured activity rather than just asking a teen what they think their triggers are would be really helpful because sometimes if teens are just asked, they won’t know but with the examples, they can begin to recognize things.” [Alpha provider] “It’s good that it teaches real facts. Most therapists, the first things they will say about them is that you should not do them, but the app actually leads you to understand why. Most of the time, a therapist will tell you that this is a very bad drug, but the app actually gives you straight facts about the drug and the effects and why it is bad rather than just saying drugs are bad and don’t do it. This educates you.” [Beta teen]
	Useful embedded resources	“I felt like the menu was pretty straightforward and liked that there was a resource section, that way if a kid is having a bad day, they can easily find someone to reach out to.” [Beta provider]
	Variety of activities	“I think it’s good to have a variety of activities as there are different times when different ones are more suitable.” [Beta teen]
	Familiarity	“It helps since teens are already comfortable with smart phones, it is something familiar whereas treatment may feel less familiar. Teens work better on their phones.” [Beta provider]
	Encourages openness and honesty	“It is useful for learning more personally...it will make them feel more comfortable answering truthfully and will be easier to answer in an app than in person, especially if you were anti-social or nervous.” [Beta teen] “App could serve as a bridge in circumstances where the teen is thinking it, but just struggling to put it into words or to say out loud. The app might also help teen feel like there is less judgment or would help them answer more honestly, particularly in circumstances where there is a parent in the room.” [Beta provider]
	User-friendly	“I think it’s extremely easy to navigate, very easy. Everything is laid out, there’s a list of activities, I knew immediately what to do.” [Beta teen] “I like that it breaks things down so you can select what is most fitting to the circumstance (cravings, resources), that makes it quicker. It’s easy to navigate through.” [Beta provider]
	Privacy or restricted access	“My only concern is parents accessing things that are private. A log in would help or facial recognition. Would want therapist to have access, but parents in general should not because you go to therapy because you can’t talk to your parents about things.” [Beta teen] “An option of a password would be a really cool feature for someone who is just trying to get help in secrecy.” [Alpha teen]
**App use in combination with outpatient therapy**		
	Improving treatment engagement	“I would like to use this in session. It would be good to start conversations with my therapist.” [Alpha teen]
	Activities outside of therapy sessions	“I think it would make a good sort of homework pre-test type thing.” [Alpha provider] “I think it would be great to give homework to clients.” [Alpha provider]
**App modifications**		
	More detailed instructions or feedback	“I’m so used to having my assignments with clear directions. I would like to see that here. I still don’t get what to do.” [Alpha provider] “I like that it gives immediate feedback and that it gives further information about the correct answer.” [Beta provider]
	Simplified language	“I don’t talk to my kid patients like this, cognitive distortions, etc. I would probably make this more at a high school reading level, like the words.” [Alpha provider]

### App Content

Providers and teens favored the use of a variety of activities and resources to provide education on topics related to substance use and mental health. Both youth and providers reported that app content was informative, developmentally appropriate, and relevant to adolescents. They found the information to be helpful regarding educating youth on various issues they could encounter and considered the app content as a healthy method for learning and coping. Many providers speculated that youth would find the information more credible than information presented by parents, other authority figures, or the internet. In addition, providers appreciated that content expanded beyond mental health and substance use by including content and activities that addressed family communication and healthy relationships.

Most participants perceived the embedded resources as useful and relevant to the purpose of the app. Providers and teens stated that having in-app access to nationally available resources is valuable for teens to locate immediate services or additional information. In addition, several participants expressed enthusiasm for the app’s inclusion of several different types of interactive modules to present information, which they perceived to be more engaging for youth than standard approaches to education in clinical settings (eg, pamphlets and explanations). Participants reported particular excitement about activities such as the *What Would You Do?* scenarios and image maps where users can click on different areas of the screen to reveal educational text or images. Several providers commented that youth might be attracted to game-like features, such as the interactive modules included in the current app.

### User Experience

Providers and youth participants noted their preference for using technology-based tools over paper documentation because of familiarity, user-friendliness, and security of apps. Several teens also recommended that app content be available to youth for self-directed use so that they could choose which activities to complete or view regardless of what may be assigned in a therapy session. A common theme across all participants was the preference for using apps instead of paper documentation to complete questionnaires and assigned activities. The participants cited convenience and accessibility of mobile apps as reasons for preferring apps. In addition, participants valued receiving immediate results and feedback following the completion of the activities.

Participants noted that youths’ familiarity with smartphones contributed to their willingness to use and interact with the app. The digital or app-based versions of certain activities were familiar and viewed as reasonable alternatives to standard care for both youth and providers. For instance, youth participants noted how the flash card–style activities mirrored physical cards a therapist may use with the added benefit of always being available, including outside of session.

Many youth participants reported that they would feel more comfortable disclosing personal information on the app than in therapy sessions. Several providers proposed that using an app in addition to verbal discussions in a session could encourage adolescent patients to be more open and candid in sharing information about their thoughts, feelings, and behaviors. Participants emphasized the importance of app privacy and suggested that there be well-defined limits on what user information is released to providers or parents. Several participants noted that using a password log-in would make teenagers feel more comfortable disclosing personal information.

Finally, the provider and teenager participants indicated that they found the app to be user-friendly and easy to navigate. Some specific app features identified as especially helpful in this regard were the search bar for navigation, embedded resources, and extensive feedback for user responses throughout the app. Many participants indicated that these features were necessary if the goal is to have youth use the app regularly.

### App Use in Combination With Outpatient Therapy

Both youth and provider participants endorsed the practicality and appropriateness of using the app in combination with outpatient treatment. They discussed ways to incorporate the app into therapy sessions (eg, to guide in-session discussions and for in-session activities) and outside of therapy as homework activities.

Several providers indicated that the app would help encourage youth patients to identify and clarify their feelings, which may prompt more productive conversations in therapy sessions and improve the youth–provider therapeutic alliance. Most youths shared this viewpoint and commented that they would feel more comfortable entering their mood or feelings into the app to collect their thoughts before discussing with their providers in therapy sessions. In addition, both youths and providers noted that the app would provide a consistent mechanism for completing treatment-related tasks in and out of sessions in a user-friendly format.

Youths and providers indicated that the app could be used for assignments to complete outside of the clinic to reinforce or assess what they learned during therapy sessions. Many youths stated they would use the app several times per week to review content and explore new activities, even if they were not assigned any homework tasks by their providers.

### App Modifications

Participants in the alpha testing phase provided suggestions for modifications to the app. Some of these recommendations were incorporated into the beta version of the app when feasible within the scope of this project. Modifications included improving the overall quality of the content and optimizing the user experience within the app. Participants in the beta testing phase also offered suggestions on improving the future version of the app.

Participants recommended using more extensive instructions and feedback for activities and generally streamlining the app functionality. Before beta testing began, detailed instruction and feedback pages were added to the activities. These additions proved to be effective as beta participants commented on easy navigation, clear instructions, and comprehensive feedback screens. Providers also preferred the simplified language in the app, allowing activities to be more approachable for youths. The simplified text incorporated more definitions throughout the app with the goal of reducing confusion and promoting understanding of key concepts.

Following demonstrations during alpha and beta testing, youths and providers reported on PU, ease of use, ease of learning, and overall satisfaction. Table 2 summarizes the scores from each cohort at each phase of the study, along with comparisons from alpha to beta testing. A significant difference was observed from alpha to beta testing in the PU of the app, whereby providers reported a slightly lower PU at beta testing relative to alpha testing. No other significant differences were observed.

**Table 2:** Youth- and provider-reported perceptions of the app at alpha and beta testing demonstration sessions (N=40).

Perceptions	Providers					Youths			
	Alpha (n=10), mean (SD)	Beta (n=10), mean (SD)	Mann–Whitney *U* test	*P* value	Alpha (n=10), mean (SD)		Beta (n=10), mean (SD)	Mann–Whitney *U* test	*P* value
Perceived usefulness	2.50 (0.26)	2.10 (0.42)	20.00	.02	2.42 (0.41)		2.33 (0.29)	41.50	.53
Perceived ease of use	2.36 (0.37)	2.34 (0.36)	45.00	.74	2.47 (0.56)		2.41 (0.31)	42.00	.58
Perceived ease of learning	2.50 (0.44)	2.45 (0.44)	48.00	.91	2.55 (0.75)		2.43 (0.41)	35.50	.28
Satisfaction	2.49 (0.28)	2.28 (0.39)	30.50	.14	2.55 (0.40)		2.23 (0.39)	29.00	.12

## Discussion

### Principal Findings

Over the past decade, there has been a proliferation in mental health apps meant to help people with behavioral health concerns manage their symptoms through coping skills, mood monitoring, and other strategies, sometimes in conjunction with formal therapy, other times as a self-directed, stand-alone app [37]. Research involving such apps has also gained considerable momentum; however, there are few apps that have been developed specifically for youth with substance use using an iterative, user-guided design process, and evidence for such tools remains sparse [38,39]. To address this gap in the clinical toolkit available to clinicians caring for youths with co-occurring substance use and mental health disorders, this mixed methods study involved user-centered design and the development of a web-based mHealth app meant to augment outpatient treatment. Specifically, this study involved an iterative process of evaluation whereby youths with behavioral health treatment experience and providers gave feedback via surveys and interviews following a live demonstration of a new mobile app.

The results indicated that both youths and providers favored the use of an mHealth app over conventional paper documentation because of several advantages of apps, including convenience, accessibility, and capacity to deliver immediate results and feedback even outside of the session. The app content was perceived to be relevant, valuable, and appropriate for the target user population of youths with behavioral health disorders. In addition, youths described feeling more comfortable disclosing personal information within the app as compared with in-person therapy. Providers noted that the use of the app alongside outpatient therapy could be especially helpful if youths complete activities outside of sessions, which would make it easier to discuss difficult topics in session or that could reinforce lessons or skills taught in session, thereby increasing the productivity and impact of treatment.

The evaluation plan was guided by the TAM, which emphasizes the importance of PU and ease of use for promoting the feasibility and acceptability of new technologies and systems. Here, ratings from both youths and providers indicated generally high (positive) ratings for both PU and PEOU, reflecting both appropriate content as well as layout and functionality. Counter to the hypotheses, there were no increased ratings of PU or PEOU from alpha testing to beta testing despite the incorporation of new content and functionality between those sessions. This is likely because of the fact that different participants were recruited for each phase (ie, beta test participants had never seen the alpha version of the app; alpha test participants were not asked to evaluate the beta version of the app). However, there was a significant difference in PU among providers, whereby beta test providers rated the app as somewhat less useful than the alpha test providers. A careful review of specific responses revealed that some beta test providers rated novelty and PU as lower because of the influx of mental health apps on the market, some of which had functionality that is not currently included in the app under investigation here (Bright Path). For instance, some providers noted that other apps include guided meditations and other recorded content to encourage coping skills [40,41], which are not currently part of the Bright Path. As clinician participants had used apps with those sorts of features with patients to enhance the positive effect of their practice, they suggested that the absence of similar features in the current iteration of Bright Path limited its potential usefulness. However, PU was rated favorably, and the critiques raised by the providers in the beta testing phase may be addressed through future software development in future versions of the app.

Participants expressed positive reactions to the variety of activity types and content presented in the app, which were derived from existing cognitive behavioral strategies for addressing substance use and other mental health disorder symptoms. Effective psychotherapy models for SUDs often include elements of motivational interviewing (MI) [42]. MI-related content was not explicitly included in the evaluated versions of the app in this study. Future versions may include more MI-consistent activities. Alternatively, the app may be deployed alongside live psychotherapy (whether in person or telehealth) where the clinician uses MI techniques in session and uses the app to deliver or augment CBT content. This approach has been used in other mHealth studies [43-45]. For instance, young adults who used an app designed to reduce cannabis use reported decreased cravings and cannabis use when delivered alongside 2 motivational enhancement therapy sessions [46]. A similar approach that leverages ecological momentary assessment and remote intervention is currently being studied with youths who exhibit dual disorders [47,48]. This work highlights the importance of considering not only *what* an app includes (content and functionality) but also *how* it is implemented with intended users.

### Limitations

The results of this study should be interpreted in light of these limitations. Although the study involved iterative testing with intended end users of the developed app, the samples of youths and providers were small at each time point, and different participants were recruited for the alpha and beta tests. Although this prevented within-subject comparisons from alpha to beta testing, which may have resulted in greater ability to detect changes in usability-related variables following edits to the app, it is still valuable to gather fresh perspectives from the intended user population. In addition, participants were recruited locally where the research was conducted, which was a large metropolitan area in the Midwestern United States (population: approximately 2 million people, including the surrounding rural and suburban communities). Perspectives on the usefulness and utility of the app may vary in different geographic regions. For instance, the relative availability of behavioral health specialists may affect the degree to which clinicians would view an app such as this as helpful in as much it might afford more opportunities for self-paced, asynchronous clinical activities so that clinicians could see more patients. Notably, the area where this work took place is a federally designated child mental health shortage area, as is much of the United States (Health Resources and Services Administration, 2021) [49], suggesting that views of youths and providers may have relevance beyond the specific community where the work was conducted. Finally, there was limited demographic diversity among clinician participants (all were women). Together, these factors may have limited the generalizability of the findings to the broader population of adolescents with substance use and mental health disorders and clinicians who provide services to youths with behavioral health disorders. A related limitation was that details about the specific SUDs and mental health disorders that the youths met the diagnostic criteria for were not collected in this study. It is possible that youths with different patterns of mental health symptoms or substance use would view or respond differently to the app. In contrast, the app was not designed to be specific to a particular substance or diagnosis and was intended to be broadly appealing and relevant to youths with SUDs and mental health comorbidities. As such, the generally positive responses and constructive feedback on actionable improvements to the app suggest that this line of research should be maintained with efforts to test future iterations of the app in a more diverse population of youths and providers.

### Conclusions

Mobile apps appear to be a promising, scalable option for supplementing existing therapeutic interventions with adolescents exhibiting co-occurring SUDs and mental health concerns. Apps can provide tools and resources to patients, permitting them to practice what they are learning in therapy in their natural environment between sessions. On the basis of the current results, youths and providers have favorable views about the potential usefulness of apps alongside outpatient therapy, including the specific app tested here, and value having a variety of developmentally appropriate activities geared toward improving treatment engagement in and out of session. Future work should continue to refine the content and functionality, as well as the implementation strategy, for maximal impact in diverse patient populations and clinical settings while also evaluating the efficacy of such tools in improving outcomes for youths with behavioral health disorders.

## Abbreviations

CBT: cognitive behavioral therapy
mHealth: mobile health
MI: motivational interviewing
PEOU: perceived ease of use
PU: perceived usefulness
SUD: substance use disorder
TAM: technology acceptance model

## References

[1] (2019). Key Substance Use and Mental Health Indicators in the United States: results from the 2018 National Survey on Drug Use and Health. Substance Abuse and Mental Health Services Administration.

[2] Trends in annual prevalence of use of various drugs. Monitoring the Future Survey.

[3] Robinson ZD, Riggs PD (2016). Cooccurring psychiatric and substance use disorders. Child Adolesc Psychiatr Clin N Am.

[4] Armstrong TD, Costello EJ (2002). Community studies on adolescent substance use, abuse, or dependence and psychiatric comorbidity. J Consult Clin Psychol.

[5] Kaminer Y, Connor DF, Curry JF (2007). Comorbid adolescent substance use and major depressive disorders: a review. Psychiatry (Edgmont).

[6] Drug use among youth: facts and statistics. National Center for Drug Abuse.

[7] Heradstveit O, Skogen JC, Hetland J, Stewart R, Hysing M (2019). Psychiatric diagnoses differ considerably in their associations with alcohol/drug-related problems among adolescents. A Norwegian population-based survey linked with national patient registry data. Front Psychol.

[8] Kelly TM, Daley DC (2013). Integrated treatment of substance use and psychiatric disorders. Soc Work Public Health.

[9] Treatment Episode Data Set (TEDS) discharges from substance abuse treatment services. Substance Abuse and Mental Health Services Administration.

[10] Henderson JL, Chaim G, Brownlie EB (2017). Collaborating with community-based services to promote evidence-based practice: process description of a national initiative to improve services for youth with mental health and substance use problems. Psychol Serv.

[11] Brewer S, Godley MD, Hulvershorn LA (2017). Treating mental health and substance use disorders in adolescents: what is on the menu?. Curr Psychiatry Rep.

[12] Shams F, Wong JS, Nikoo M, Outadi A, Moazen-Zadeh E, Kamel MM, Song MJ, Jang KL, Krausz RM (2021). Understanding ehealth cognitive behavioral therapy targeting substance use: realist review. J Med Internet Res.

[13] Vujanovic AA, Meyer TD, Heads AM, Stotts AL, Villarreal YR, Schmitz JM (2017). Cognitive-behavioral therapies for depression and substance use disorders: an overview of traditional, third-wave, and transdiagnostic approaches. Am J Drug Alcohol Abuse.

[14] Carroll KM, Kiluk BD (2017). Cognitive behavioral interventions for alcohol and drug use disorders: through the stage model and back again. Psychol Addict Behav.

[15] Kazantzis N, Brownfield NR, Mosely L, Usatoff AS, Flighty AJ (2017). Homework in cognitive behavioral therapy: a systematic review of adherence assessment in anxiety and depression (2011-2016). Psychiatr Clin North Am.

[16] Darnell D, O'Connor S, Wagner A, Russo J, Wang J, Ingraham L, Sandgren K, Zatzick D (2017). Enhancing the reach of cognitive-behavioral therapy targeting posttraumatic stress in acute care medical settings. Psychiatr Serv.

[17] Bunnell BE, Nemeth LS, Lenert LA, Kazantzis N, Deblinger E, Higgins KA, Ruggiero KJ (2021). Barriers associated with the implementation of homework in youth mental health treatment and potential mobile health solutions. Cognit Ther Res.

[18] Gaynor ST, Lawrence PS, Nelson-Gray RO (2006). Measuring homework compliance in cognitive-behavioral therapy for adolescent depression: review, preliminary findings, and implications for theory and practice. Behav Modif.

[19] Wyatt TH, Bayless AK, Krauskopf P, Gaylord N (2021). Using mHealth applications to promote self-managed health behaviors among teens. J Pediatr Nurs.

[20] Marsch LA (2012). Leveraging technology to enhance addiction treatment and recovery. J Addict Dis.

[21] Most US teens who use cellphones do it to pass time, connect with others, learn new things. Pew Research Center.

[22] Curtis BL, Ashford RD, Magnuson KI, Ryan-Pettes SR (2019). Comparison of smartphone ownership, social media use, and willingness to use digital interventions between generation z and millennials in the treatment of substance use: cross-sectional questionnaire study. J Med Internet Res.

[23] Donker T, Petrie K, Proudfoot J, Clarke J, Birch M, Christensen H (2013). Smartphones for smarter delivery of mental health programs: a systematic review. J Med Internet Res.

[24] Hutton A, Prichard I, Whitehead D, Thomas S, Rubin M, Sloand E, Powell TW, Frisch K, Newman P, Goodwin Veenema T (2020). mHealth interventions to reduce alcohol use in young people: a systematic review of the literature. Compr Child Adolesc Nurs.

[25] Mason M, Ola B, Zaharakis N, Zhang J (2015). Text messaging interventions for adolescent and young adult substance use: a meta-analysis. Prev Sci.

[26] Marsch LA, Borodovsky JT (2016). Technology-based interventions for preventing and treating substance use among youth. Child Adolesc Psychiatr Clin N Am.

[27] Fedele DA, Cushing CC, Fritz A, Amaro CM, Ortega A (2017). Mobile health interventions for improving health outcomes in youth: a meta-analysis. JAMA Pediatr.

[28] (2019). ISO 9241-210:2019 Ergonomics of human-system interaction — Part 210: human-centred design for interactive systems. International Organization for Standardization.

[29] Schnall R, Rojas M, Bakken S, Brown W, Carballo-Dieguez A, Carry M, Gelaude D, Mosley JP, Travers J (2016). A user-centered model for designing consumer mobile health (mHealth) applications (apps). J Biomed Inform.

[30] (2017). Mobile Health: Sensors, Analytic Methods, and Applications.

[31] Garrido S, Cheers D, Boydell K, Nguyen QV, Schubert E, Dunne L, Meade T (2019). Young people's response to six smartphone apps for anxiety and depression: focus group study. JMIR Ment Health.

[32] Knight JR, Sherritt L, Shrier LA, Harris SK, Chang G (2002). Validity of the CRAFFT substance abuse screening test among adolescent clinic patients. Arch Pediatr Adolesc Med.

[33] Mitchell SG, Kelly SM, Gryczynski J, Myers CP, O'Grady KE, Kirk AS, Schwartz RP (2014). The CRAFFT cut-points and DSM-5 criteria for alcohol and other drugs: a reevaluation and reexamination. Subst Abus.

[34] Shenoi RP, Linakis JG, Bromberg JR, Casper TC, Richards R, Mello MJ, Chun TH, Spirito A, PEDIATRIC EMERGENCY CARE APPLIED RESEARCH NETWORK (2019). Predictive validity of the CRAFFT for substance use disorder. Pediatrics.

[35] A technology acceptance model for empirically testing new end-user information systems: theory and results. Massachusetts Institute of Technology.

[36] Solomon P (1995). The think aloud method: a practical guide to modelling cognitive processes. Inf Process Manag.

[37] Jaspers MW, Steen T, van den Bos C, Geenen M (2004). The think aloud method: a guide to user interface design. Int J Med Inform.

[38] Xu W, Liu Y (2015). mHealthApps: a repository and database of mobile health apps. JMIR Mhealth Uhealth.

[39] Linardon J, Cuijpers P, Carlbring P, Messer M, Fuller-Tyszkiewicz M (2019). The efficacy of app-supported smartphone interventions for mental health problems: a meta-analysis of randomized controlled trials. World Psychiatry.

[40] Nunes A, Castro SL, Limpo T (2020). A review of mindfulness-based apps for children. Mindfulness.

[41] Hutchinson E (2020). Mindfulness apps. School Librarian.

[42] Help me find an app. One Mind Psyber Guide.

[43] Adam A, Jain A, Pletnikova A, Bagga R, Vita A, N Richey L, Gould N, Munshaw S, Misrilall K, Peters ME (2020). Use of a mobile app to augment psychotherapy in a community psychiatric clinic: feasibility and fidelity trial. JMIR Form Res.

[44] Ben-Zeev D, Drake RE, Corrigan PW, Rotondi AJ, Nilsen W, Depp C (2012). Using contemporary technologies in the assessment and treatment of serious mental illness. Am J Psychiatric Rehab.

[45] Wilansky P, Eklund JM, Milner T, Kreindler D, Cheung A, Kovacs T, Shooshtari S, Astell A, Ohinmaa A, Henderson J, Strauss J, Mills RS (2016). Cognitive behavior therapy for anxious and depressed youth: improving homework adherence through mobile technology. JMIR Res Protoc.

[46] Hogue A, Henderson CE, Becker SJ, Knight DK (2018). Evidence base on outpatient behavioral treatments for adolescent substance use, 2014-2017: outcomes, treatment delivery, and promising horizons. J Clin Child Adolesc Psychol.

[47] Pennou A, Lecomte T, Potvin S, Khazaal Y (2019). Mobile intervention for individuals with psychosis, dual disorders, and their common comorbidities: a literature review. Front Psychiatry.

[48] Benarous X, Edel Y, Consoli A, Brunelle J, Etter J, Cohen D, Khazaal Y (2016). Ecological momentary assessment and smartphone application intervention in adolescents with substance use and comorbid severe psychiatric disorders: study protocol. Front Psychiatry.

[49] Shortage areas. Human Resource & Services Administration.

